# Supplementary material for: Pyruvate Dehydrogenase Kinase 1 inhibition mediated oxidative phosphorylation enhancement in cartilage promotes osteoarthritis progression
Source: BMC Musculoskelet Disord. 2023 Jul 20;24:597. doi: 10.1186/s12891-023-06585-6 (PMC10357736; doi:10.1186/s12891-023-06585-6)
Supplement: Supplementary file 1 — Additional file 1. [file 12891_2023_6585_MOESM1_ESM.zip › KEGG.Gsea.1653622432464/KEGG_ALDOSTERONE_REGULATED_SODIUM_REABSORPTION.html]

Details for gene set KEGG\_ALDOSTERONE\_REGULATED\_SODIUM\_REABSORPTION[GSEA]

|  || Dataset | 55457\_collapsed\_to\_symbols.cls.cls #OA\_versus\_normal.cls.cls #OA\_versus\_normal\_repos |
| Phenotype | cls.cls#OA\_versus\_normal\_repos |
| Upregulated in class | normal |
| GeneSet | KEGG\_ALDOSTERONE\_REGULATED\_SODIUM\_REABSORPTION |
| Enrichment Score (ES) | -0.32204714 |
| Normalized Enrichment Score (NES) | -1.1604251 |
| Nominal p-value | 0.24531516 |
| FDR q-value | 0.433609 |
| FWER p-Value | 1.0 |
Table: GSEA Results Summary

  

Fig 1: Enrichment plot: KEGG\_ALDOSTERONE\_REGULATED\_SODIUM\_REABSORPTION      
 Profile of the Running ES Score & Positions of GeneSet Members on the Rank Ordered List

  

| SYMBOL | TITLE | RANK IN GENE LIST | RANK METRIC SCORE | RUNNING ES | CORE ENRICHMENT || 1 | HSD11B2 | hydroxysteroid 11-beta dehydrogenase 2 [Source:HGNC Symbol;Acc:HGNC:5209] | 539 | 0.645 | 0.0126 | No |
| 2 | MAPK3 | mitogen-activated protein kinase 3 [Source:HGNC Symbol;Acc:HGNC:6877] | 757 | 0.579 | 0.0446 | No |
| 3 | ATP1B1 | ATPase Na+/K+ transporting subunit beta 1 [Source:HGNC Symbol;Acc:HGNC:804] | 1177 | 0.481 | 0.0526 | No |
| 4 | PIK3CB | "phosphatidylinositol-4,5-bisphosphate 3-kinase catalytic subunit beta [Source:HGNC Symbol;Acc:HGNC:8976]" | 2186 | 0.340 | 0.0029 | No |
| 5 | IGF1 | insulin like growth factor 1 [Source:HGNC Symbol;Acc:HGNC:5464] | 2286 | 0.328 | 0.0230 | No |
| 6 | HSD11B1 | hydroxysteroid 11-beta dehydrogenase 1 [Source:HGNC Symbol;Acc:HGNC:5208] | 3000 | 0.250 | -0.0114 | No |
| 7 | NR3C2 | nuclear receptor subfamily 3 group C member 2 [Source:HGNC Symbol;Acc:HGNC:7979] | 3335 | 0.218 | -0.0190 | No |
| 8 | PRKCA | protein kinase C alpha [Source:HGNC Symbol;Acc:HGNC:9393] | 3728 | 0.183 | -0.0340 | No |
| 9 | NEDD4L | NEDD4 like E3 ubiquitin protein ligase [Source:HGNC Symbol;Acc:HGNC:7728] | 3733 | 0.183 | -0.0188 | No |
| 10 | INS | insulin [Source:HGNC Symbol;Acc:HGNC:6081] | 3885 | 0.166 | -0.0166 | No |
| 11 | FXYD2 | FXYD domain containing ion transport regulator 2 [Source:HGNC Symbol;Acc:HGNC:4026] | 4073 | 0.149 | -0.0186 | No |
| 12 | ATP1A3 | ATPase Na+/K+ transporting subunit alpha 3 [Source:HGNC Symbol;Acc:HGNC:801] | 5475 | 0.035 | -0.1246 | No |
| 13 | IRS4 | insulin receptor substrate 4 [Source:HGNC Symbol;Acc:HGNC:6128] | 6106 | -0.013 | -0.1724 | No |
| 14 | PRKCB | protein kinase C beta [Source:HGNC Symbol;Acc:HGNC:9395] | 6453 | -0.039 | -0.1961 | No |
| 15 | MAPK1 | mitogen-activated protein kinase 1 [Source:HGNC Symbol;Acc:HGNC:6871] | 6853 | -0.068 | -0.2214 | No |
| 16 | SCNN1A | sodium channel epithelial 1 subunit alpha [Source:HGNC Symbol;Acc:HGNC:10599] | 7216 | -0.097 | -0.2413 | No |
| 17 | ATP1B4 | ATPase Na+/K+ transporting family member beta 4 [Source:HGNC Symbol;Acc:HGNC:808] | 7306 | -0.103 | -0.2396 | No |
| 18 | PIK3CD | "phosphatidylinositol-4,5-bisphosphate 3-kinase catalytic subunit delta [Source:HGNC Symbol;Acc:HGNC:8977]" | 7405 | -0.111 | -0.2378 | No |
| 19 | PIK3CA | "phosphatidylinositol-4,5-bisphosphate 3-kinase catalytic subunit alpha [Source:HGNC Symbol;Acc:HGNC:8975]" | 7733 | -0.136 | -0.2517 | No |
| 20 | PIK3CG | "phosphatidylinositol-4,5-bisphosphate 3-kinase catalytic subunit gamma [Source:HGNC Symbol;Acc:HGNC:8978]" | 7953 | -0.152 | -0.2560 | No |
| 21 | SFN | stratifin [Source:HGNC Symbol;Acc:HGNC:10773] | 8118 | -0.164 | -0.2549 | No |
| 22 | ATP1A2 | ATPase Na+/K+ transporting subunit alpha 2 [Source:HGNC Symbol;Acc:HGNC:800] | 8218 | -0.173 | -0.2480 | No |
| 23 | ATP1A1 | ATPase Na+/K+ transporting subunit alpha 1 [Source:HGNC Symbol;Acc:HGNC:799] | 8282 | -0.177 | -0.2379 | No |
| 24 | PRKCG | protein kinase C gamma [Source:HGNC Symbol;Acc:HGNC:9402] | 8582 | -0.200 | -0.2443 | No |
| 25 | PIK3R3 | phosphoinositide-3-kinase regulatory subunit 3 [Source:HGNC Symbol;Acc:HGNC:8981] | 8801 | -0.217 | -0.2429 | No |
| 26 | PIK3R5 | phosphoinositide-3-kinase regulatory subunit 5 [Source:HGNC Symbol;Acc:HGNC:30035] | 8804 | -0.217 | -0.2248 | No |
| 27 | SLC9A3R2 | SLC9A3 regulator 2 [Source:HGNC Symbol;Acc:HGNC:11076] | 8831 | -0.220 | -0.2082 | No |
| 28 | SCNN1B | sodium channel epithelial 1 subunit beta [Source:HGNC Symbol;Acc:HGNC:10600] | 8945 | -0.227 | -0.1978 | No |
| 29 | KRAS | "KRAS proto-oncogene, GTPase [Source:HGNC Symbol;Acc:HGNC:6407]" | 10544 | -0.377 | -0.2902 | Yes |
| 30 | ATP1B2 | ATPase Na+/K+ transporting subunit beta 2 [Source:HGNC Symbol;Acc:HGNC:805] | 10595 | -0.383 | -0.2617 | Yes |
| 31 | PIK3R2 | phosphoinositide-3-kinase regulatory subunit 2 [Source:HGNC Symbol;Acc:HGNC:8980] | 10956 | -0.425 | -0.2538 | Yes |
| 32 | PIK3R1 | phosphoinositide-3-kinase regulatory subunit 1 [Source:HGNC Symbol;Acc:HGNC:8979] | 11099 | -0.443 | -0.2274 | Yes |
| 33 | IRS2 | insulin receptor substrate 2 [Source:HGNC Symbol;Acc:HGNC:6126] | 11390 | -0.485 | -0.2090 | Yes |
| 34 | KCNJ1 | potassium inwardly rectifying channel subfamily J member 1 [Source:HGNC Symbol;Acc:HGNC:6255] | 11418 | -0.489 | -0.1698 | Yes |
| 35 | ATP1B3 | ATPase Na+/K+ transporting subunit beta 3 [Source:HGNC Symbol;Acc:HGNC:806] | 11424 | -0.490 | -0.1288 | Yes |
| 36 | PDPK1 | 3-phosphoinositide dependent protein kinase 1 [Source:HGNC Symbol;Acc:HGNC:8816] | 11839 | -0.557 | -0.1139 | Yes |
| 37 | IRS1 | insulin receptor substrate 1 [Source:HGNC Symbol;Acc:HGNC:6125] | 11841 | -0.557 | -0.0669 | Yes |
| 38 | SGK1 | serum/glucocorticoid regulated kinase 1 [Source:HGNC Symbol;Acc:HGNC:10810] | 12357 | -0.694 | -0.0483 | Yes |
| 39 | INSR | insulin receptor [Source:HGNC Symbol;Acc:HGNC:6091] | 12856 | -1.071 | 0.0034 | Yes |
Table: GSEA details [plain text format]

  

Fig 2: KEGG\_ALDOSTERONE\_REGULATED\_SODIUM\_REABSORPTION      
 Blue-Pink O' Gram in the Space of the Analyzed GeneSet

  

Fig 3: KEGG\_ALDOSTERONE\_REGULATED\_SODIUM\_REABSORPTION: Random ES distribution      
 Gene set null distribution of ES for **KEGG\_ALDOSTERONE\_REGULATED\_SODIUM\_REABSORPTION**
